# Understanding the barriers and factors to HIV testing intention of women engaging in compensated dating in Hong Kong: The application of the extended Theory of Planned Behavior

**DOI:** 10.1371/journal.pone.0213920

**Published:** 2019-06-27

**Authors:** Phoenix K. H. Mo, Joseph T. F. Lau, Meiqi Xin, Vivian W. I. Fong

**Affiliations:** School of Public Health and Primary Care, The Chinese University of Hong Kong, Hong Kong; Qazvin University of Medical Sciences, ISLAMIC REPUBLIC OF IRAN

## Abstract

**Background:**

Women engaging in compensated dating is one of the at risk group of HIV infection due to multiple sexual partnerships and risky sexual practices. The present study examined the prevalence of HIV testing behavior and intention, and identified factors associated with HIV testing intention among women engaging in compensated dating in Hong Kong. Factors from the Theory of Planned Behaviors and the role of various types of barriers to HIV testing were also explored.

**Methods:**

An anonymous, cross-sectional survey was conducted online. Target participants were women who have engaged in compensated dating and provided sex services to clients. Participants were recruited via three sources, including i) online outreaching, ii) referral made by NGOs, and iii) referral made by participants. A total of 183 participants completed the study.

**Results:**

Respectively 29.7% and 18.6% have taken up HIV testing and showed intention to take up HIV testing in the future year. Results from the multiple hierarchical regression model showed that having ever received HIV testing (β = 0.44, p < .001), attitudes towards HIV testing (β = 0.22, p < .01), subjective norm (β = 0.18, p < .05), perceived behavioral control (β = 0.18, p < .01) and perceived discrimination from health care workers (β = -0.24, p < .05) significantly predicted intention to take up HIV testing.

**Conclusion:**

The present study demonstrates the applicability of extended TPB in HIV testing intention among women engaging in compensated dating. Interventions to promote HIV testing among this group are greatly warranted.

## Introduction

Compensated dating, also known as Enjo-kōsai, is a type of transactional relationship that involves providing compensation, usually financial remuneration, to young women for their companionship or possibly for sexual favors [1]. Many women usually start compensated dating off in relationships that only involve emotional companionship but eventually provide sexual services without any coercion. Most of the women engaging in compensated dating are secondary or college students and mainly seek clients online [2]. In Hong Kong, a steady increase in young women entering this line of work is observed in the recent years. One cross-sectional telephone-based survey with 1,010 12-29-year-old youth in Hong Kong found that the lifetime prevalence of compensated dating was 2.7% [3]. Another local study among 3,638 grade 8 students (mean age = 13.6 years old) also reported that around 3% of the participants have ever engaged in compensated dating [4]. Approximately 70% of the compensated dating involved sex transaction [5]. Compensated dating has been considered a new and unique trend of sex work and an important public health concern in Hong Kong [6].

### Women engaging in compensated dating as important at risk group for HIV infection

Women engaging in compensated dating is one of the at risk groups of HIV infection due to their multiple sexual partnerships and risky sexual practices [7]. One local study found that compensated dating was significantly associated with various types of sex-related risk behaviors including unsafe sex, infection of sexually transmitted diseases (STDs), unintended pregnancy, and abortion [3]. In Hong Kong, heterosexual HIV transmission constitutes of 38.8% of all HIV infections. [8]. Service provision to this at risk group is difficult as most of them are highly mobile and marginalized with most of their deals being negotiated over the Internet [9].

### HIV testing as an important HIV prevention strategy

HIV testing is one of the recommended strategies for HIV care and prevention [10]. HIV testing may help individuals who are exposed to the risk of HIV infection know their HIV serostatus so that early treatment can be provided and further HIV transmission can be avoided. Locally, the prevalence of HIV testing was only 9.3% and 30.6%, among female sex workers (FSW) recruited from the Internet and Karaoke respectively [11]. The low level of HIV testing uptake was consistent with those reported from the Hong Kong Department of Health (i.e. about half took up HIV testing in the past year) [12], and from other countries (e.g. 20% to 50% of FSW reported prior HIV testing) [13–15]. Currently, there is a lack of data on the HIV testing rate of women engaging in compensated dating in Hong Kong. As most of the women engaging in compensated dating are generally younger, they may face certain young-age-specific barriers, i.e. fear of disclosing their sexual activity to parents/guardian/health workers, which may result in lower intention to engage in HIV testing [16]. Together with the limited access to HIV-related information and services, women engaging in compensated dating are expected to have lower HIV testing rate compared to other FSW counterparts. A report from Global Network of Sex Work Projects also concluded that young female sex workers were often excluded from their services that promoted HIV testing [16].

### Factors to uptake of HIV testing

Previous studies have reported a number of factors associated with uptake of HIV testing among FSWs, including sexual risk behaviors [17] such as decreased condom use [18] and recent drug use [14, 17], high perceived risk of HIV [14], and having regular health care provider or clinic [17]. Likewise, the application of behavioral health models in understanding testing behavior is important for researchers to identify possible mechanisms through which HIV testing can be promoted. Among the health models, the Theory of Planned Behavior (TPB) [19] has been one of the most widely investigated. The TPB asserts that intention to engage in a behavior is the proximal determinants of behavior. Three major components, namely, attitudes, subjective norm, and perceived behavioral control, are important in explaining behavioral intention and subsequently the behavior. According to the TPB, individuals would have higher intention to engage in a behavior if the overall evaluation of the behavior is positive (attitudes), if they believe that people they value think they should perform the behavior (subjective norm), and if they feel that they have the necessary control in performing the anticipated behavior (perceived behavioral control).

TPB has been widely applied and found to be effective in understanding a variety of health behaviors, including help-seeking behavior [20, 21], physical activity [22], dietary behaviors [23] and alcohol consumption [20, 24]. In the context of HIV testing, some studies found that among the TPB variables, subjective norms and attitudes were significant in predicting intention to use HIV counselling and testing among health professionals [25]. Alternatively, other studies showed that attitudes and perceived behavioral control were significant in predicting HIV testing intention among Tanzanian teachers [26], and Nigerian university students [27]. Moreover, a meta-analysis found that TPB accounted for 39% and 27% of the variance in intention and behaviors, providing further evidence to the effectiveness of the theory [28]. Nevertheless, no studies have used the TPB in explaining HIV testing intention among women engaging in compensated dating.

Despite its wide applicability, the TPB has been criticized for being too parsimonious that it should be modified to fit into various populations or situations. Some studies have proposed an extended TPB [29, 30] to take account into other factors that may also be important in predicting a behavior. To produce a more thorough explanation on how women engaging in compensated dating may take up HIV testing, the present study proposed to include perceived barriers to form the extended TPB. Access to HIV services could be hindered by various types of barriers, it is therefore important to understand how different types of barriers would affect one’s intention to take up HIV testing. One study among FSWs in India have documented that fear of positive HIV test results and fear of disclosure of sex work were key personal and interpersonal barriers, while experiences of being discriminated by healthcare professionals, perceived discriminatory attitudes of staff, concerns about maintaining confidentiality, poor physical facilities, non-availability of services, and long waiting times were structural barriers to assessing HIV services [13]. Other studies have also echoed that fear of social stigma after receiving the HIV positive results, and judgmental attitudes of health care workers were important barriers to HIV testing among FSWs [31, 32].

### The present study

The present study examined the prevalence of HIV testing behavior and intention, and identified factors associated with HIV testing intention among women engaging in compensated dating in Hong Kong. Utilizing the extended TPB, attitudes toward HIV testing, subjective norm, and perceived behavioral control were chosen as the potential factors. The role of various types of barriers to HIV testing was also explored.

## Methodology

### Study design

An anonymous, cross-sectional online survey was conducted between April 2017 to February 2018. The online survey was placed in a self-developed website. Online surveys have been frequently used in HIV-related research [33, 34]. It can access the hard-to-reach population effectively. It can also reduce embarrassments and interviewer bias as the study is anonymous. Target participants were women who have engaged in compensated dating and provided sex services to clients. Inclusion criteria were: i) female, ii) aged between 18 to 24, iii) has engaged in commercial sex in the past six months, which was defined as an exchange of money or gift for a sex trade, iv) have recruited sex clients through the internet (website or online apps), v) ability to read Chinese. Ethical approval of this study was obtained from The Survey and Behavioral Research Ethics Committee of the Chinese University of Hong Kong.

### Participant recruitment

Participants were recruited via three sources, including i) online outreaching, ii) referral made by NGOs, and iii) referral made by participants. For the online outreaching, women engaging in compensated dating were identified through the most common websites and online apps for seeking these women in Hong Kong. These websites and apps contain the contact information of those women who seek to recruit clients online. Electronic personal messages were sent to prospective participants to introduce the study and invite them to take part in the study. After confirming participants’ eligibility, a link of the online survey was sent to the prospective participants. Informed consent was sought online by clicking the “I agree” button before the study began. Those who completed the survey received an incentive of HKD50 upon the provision of a valid mailing address. Such incentive was mailed to them.

For referrals made by NGOs, participants were recruited by staff from local NGOs which provided HIV preventive services to women engaging in compensated dating in Hong Kong. The staff approached prospective participants when they attended their HIV preventive services, and invited them to participate in the study after confirming their eligibility. Participants were informed that participation was voluntary and refusal would not affect their right to use any services of the NGO. After confirming participants’ eligibility, a link of the online survey was sent to the prospective participants. Participants accessed the online survey and provided informed consent before they took part in the online survey. An incentive of HKD50 was given by the staff upon completion.

For referral made by participants, those who have completed the online survey would receive a participant referral number and were asked to further invite a maximum of three peers to take part in the study. The interested peers would be contacted by our research assistants. The aforementioned procedure for establishing eligibility, briefing and consent was used.

### Measures

Socio-demographic and background characteristics, including age, education level, duration of engaging in compensated dating, STD history, and frequency of unprotected sex in the past 6 month, were collected.

HIV testing. Participants were asked if they have ever taken HIV testing. They were also asked to indicate their intention to take up HIV testing in the following 12 months on a 5-point Likert scale from 1 = very unlikely to 5 = very likely.

Cognitions about HIV testing based on the TPB. Cognitions about HIV testing were measured based on the TPB Questionnaire [35]. It measures the following constructs: attitudes towards HIV testing (2 items), subjective norm towards HIV testing (2 items), and perceived behavioral control towards HIV testing (1 item). Items were rated on a 5-point Likert scale from 1 = strongly disagree to 5 = strongly agree, with higher score indicating higher level of agreement to the respective dimension. The reliability of the items was satisfactory (Cronbach’s α = .75 and 0.65 for attitudes and subjective norm respectively).

Barriers to HIV testing. Items on barriers to HIV testing were developed based on the literature on the barriers to HIV testing among FSWs [13, 31, 32], as well as discussions made between the authors and staff at the collaborating NGOs which provide HIV preventive services to women engaging in compensated dating. The items measure 4 dimensions of barriers: perceived discrimination from workers (1 item), negative feelings about HIV testing (4 items), concerns about privacy of HIV testing (3 items), and structural barriers (2 items). Items were rated on a 5-point Likert scale from 1 = strongly disagree to 5 = strong agree, with higher score indicating higher level of agreement to the respective dimension. The reliability of the items was satisfactory (Cronbach’s α ranged from 0.82 to 0.88).

### Analytic strategies

Descriptive statistics of the studied variables and pearson’s correlations between the studied variables were presented. Multiple hierarchical linear regression analyses were performed to identify the factors associated with intention to take up HIV testing. Demographic and background variables were entered into Block 1, followed by variables related to TPB (i.e. attitudes, subjective norm, perceived behavioral control) in Block 2, and variables involving barriers to HIV testing (i.e., perceived discrimination from workers, negative feelings about HIV testing, concerns about privacy, and structural barriers) in Block 3. Analyses were conducted using SPSS Version 21.0 and variables with p < .05 were considered statistically significant.

## Results

### Descriptive statistics

Table 1 shows the descriptive statistic of the participants’ sociodemographic and background characteristics. A total of 183 participants completed the online survey. The mean age of participants was 21.17 years old (SD = 0.24). Majority of them (94.0%) were single and more than two-third (72.7%) of them have attained University level of education or above. Nearly half of the participants (45.4%) have engaged in compensated dating for less than 6 months, 74.9% reported they have engaged in unprotected sex in the past 6 months. About one-tenth (10.4%) have been diagnosed with STD before and less than one third (30.1%) have ever received HIV testing. Less than a quarter (18.6%) reported that they had the intention to take up HIV testing in the following 12 months.

**Table 1:** Socio-demographic and background characteristics of participants (N = 183).

	N(%) / Mean (SD)
**Age**	Mean = 21.17, SD = 0.24
**Marital status**	
Single	172 (94%)
Cohabitating with boyfriend	9 (4.9%)
Divorced/ separated	2 (1.0%)
**Level of education**	
Primary or below	6 (3.3%)
Secondary	41 (20.4%)
University or above	133 (72.7%)
**Duration of compensated dating**	
Less than 6 months	83 (45.4%)
6 to 12 months	31 (16.9%)
13 to 24 months	40 (21.9%)
25 to 60 months	20 (10.9%)
61 months or above	9 (4.9%)
**Unprotected sex in the past 6 months**	137 (74.9%)
**History of STD**	19 (10.4%)
**Ever received HIV testing**	55 (30.1%)
**Intention to take up HIV testing in the following 12 months**	
Very unlikely	48 (26.2%)
Unlikely	77 (42.1%)
Moderate	24 (13.1%)
Likely	20 (10.9%)
Very likely	14 (7.7%)

Table 2 shows the mean score of the TPB variables and barriers to HIV testing. Among the TPB variables, items related to attitudes had the highest score (M = 3.70, SD = 0.97), followed by perceived behavioral control (M = 2.54, SD = 0.80) and subjective norm (M = 2.70, SD = 0.74). Among the various barriers of HIV testing, negative feelings about HIV testing had the highest score (M = 3.54, SD = 0.99), followed by concern about privacy (M = 3.28, SD = 1.05), perceived discrimination from workers (M = 3.17, SD = 1.23), and structural barriers (M = 3.07, SD = 1.02).

**Table 2:** Mean score of TPB variables and barriers to HIV testing (N = 183).

	Mean (SD)
**Attitudes towards HIV testing (α = 0.75)**	3.70 (0.97)
Taking up HIV testing can give you a peace of mind.	3.68 (1.08)
If you have HIV, taking up HIV testing allows you to detect HIV and seek early treatment	3.73 (1.08)
**Subjective norm (α = 0.65)**	2.70 (0.74)
The people who are important to you will support you to take up HIV testing.	2.82 (0.87)
From our understanding, how many women engaging in compensated dating in Hong Kong have received HIV testing?	2.54 (0.80)
**Perceived behavioral control**	
Taking up HIV testing or not is under your control	3.46 (0.98)
**Barriers: Perceived discrimination from workers**	
Health care workers will discriminate against you if you take up HIV testing.	3.17 (1.23)
**Barriers: negative feelings about HIV testing (α = 0.86)**	3.54 (0.99)
Taking up HIV testing makes you feel embarrassed	3.36 (1.21)
The process of HIV testing makes you feel nervous	3.45 (1.10)
You feel anxious when waiting for the HIV testing result	3.56 (1.13)
You are fear of the possibility of getting a positive result	3.77 (1.18)
**Barriers: Concern about privacy (α = 0.82)**	3.28 (1.05)
You are worried that workers will ask you very personal questions	3.43 (1.17)
You are worried about confidentiality of the HIV testing result	3.32 (1.21)
You are worried that taking HIV testing will reveal that you have engaged in compensated dating	3.11 (1.28)
**Barriers: Structural barriers (α = 0.88)**	3.07 (1.02)
There isn’t a convenient time for you to do HIV testing.	3.09 (1.10)
The venue for HIV testing is not convenient	3.04 (1.04)

### Correlation between variables

Table 3 shows the correlation between the variables under studied. Among the sociodemographic and background variables, intention to take up HIV testing was negatively correlated with education level (r = -0.18, p < .01) and positively correlated with duration of compensated dating (r = 0.15, p < .05), history of STD (r = 0.19, p < .01), and having ever received HIV testing (r = 0.53, p < .001). Intention to take up HIV testing was also significantly correlated with all three TPB variables (r = 0.27, p < .001 for attitudes, r = 0.25, p < .001 for subjective norm, and r = 0.17, p < .05 for perceived behavioral control).

**Table 3:** Correlation between variables (N = 183).

	1	2	3	4	5	6	7	8	9	10	11	12	13	14	15
1. Intention to take up HIV testing	-														
2. Age	0.03	-													
3. Marital Status	-0.03	0.01	-												
4. Education level	-0.18**	0.07	-0.01	-											
5. Duration of compensated dating	0.15*	0.12	0.15*	-0.10	-										
6. Unprotected sex over last 6 months	-0.02	0.04	-0.02	-0.09	-0.02	-									
7. History of STD	0.19**	0.11	0.16*	-0.04	0.15*	0.09	-								
8. Ever received HIV testing	0.53***	0.13	0.10	-0.15*	0.21**	0.03	0.33***	-							
9. Attitudes	0.27***	-0.03	-0.01	0.08	-0.03	-0.06	0.12	0.13*	-						
10. Subjective norm	0.25***	-0.02	-0.10	0.05	-0.09	0.06	0.00	0.12	0.34***	-					
11. Perceived behavioral control	0.17*	-0.09	0.03	0.03	-0.07	-0.01	-0.43	-0.10	0.33***	0.21**	-				
12. Barriers: Perceived discrimination from workers	-0.06	0.02	-0.10	0.03	-0.04	-0.06	-0.10	-0.06	-0.29***	-0.28***	0.08	-			
13. Barriers: Negative feelings about HIV testing	0.11	-0.01	-0.07	0.09	-0.07	-0.05	0.07	0.08	-0.59***	-0.30***	-0.24***	0.66***	-		
14. Barriers: Concern about privacy	0.08	-0.05	-0.09	0.01	-0.01	-0.35	-0.05	0.02	-0.30***	-0.23***	-0.13*	0.59***	0.57***	-	
15. Barriers: Structural barriers	0.09	0.10	-0.07	0.04	-0.14*	0.01	-0.72	-0.04	-0.26***	-0.35***	-0.12	0.57***	0.48***	0.60***	-

*p < .05

**p < .01

***p<0.001

### Multiple hierarchical regression model of intention to take up HIV testing

Table 4 shows the results of the multiple hierarchical regression models. In the first block, only having ever received HIV testing showed a significant relationship with intention to take up HIV testing (β = 0.50, p < .001). It remained significant (β = 0.40, p < .001) when variables related to TPB were entered in the second black. In the second block, attitudes toward HIV testing (β = 0.21, p < .01), subjective norm, (β = 0.17, p < .05), and perceived behavioral control (β = 0.16, p < .05) all significantly predicted intention to take up HIV testing. In the third block when various types of barriers were entered, having ever received HIV testing (β = 0.44, p < .001), attitudes towards HIV testing (β = 0.24, p < .01), subjective norm (β = 0.17, p < .05) and perceived behavioral control (β = 0.18, p < .01) remained significant. Among the barriers, only perceived discrimination from health care workers (β = -0.24, p < .05) significantly predicted intention to take up HIV testing. The final regression model explained 42% of the variance in intention to take up HIV testing, F (14,156) = 8.06, p < .001.

**Table 4:** Multiple hierarchical regression model on factors associated with intention to take up HIV testing (N = 183).

	Block 1 β	Block 2 β	Block 3 β
Age	-0.40	-0.03	-0.04
Marital Status	-0.09	-0.06	-0.07
Educational level	-0.11	-0.14	-0.14
Sex work Duration	0.04	0.06	0.07
Unprotected sex over the past 6 months	-0.05	-0.04	-0.07
History of STD	0.04	0.02	0.01
Ever received HIV testing	0.50***	0.45***	0.44***
Attitudes		0.21**	0.24**
Subjective norm		0.17*	0.17*
Perceived behavioral control		0.16*	0.18**
Barriers–Perceived discrimination from workers			-0.24*
Barriers–Negative feelings about HIV testing			0.02
Barriers–Concerns about privacy			0.04
Barriers: Structural barriers			0.14
R^2^	0.30	0.39	0.42
Df	7/163	10/160	14/156
F	10.04***	10.03***	8.06***

*p < .05

**p < .01

***p<0.001

## Discussion

Compensated dating has become an important public health concern due to its strong association with a range of negative correlates including unsafe sex, increased STD and HIV risk. Furthermore, women engaging in compensated dating are one of the few marginalized groups in Hong Kong. To date, there is limited research in this area. Very little is known about their HIV testing behaviors, and factors associated with HIV testing. A huge knowledge gap exists. Such data are largely warranted for effective service planning and provision to this vulnerable group.

Although women who entered prostitution often have a relatively lower level of education, it is interesting to note that over 70% of the current sample had university level of education or above. As the present study targeted women who were 18 years old were older, it could be conceivable that a majority of them would have achieved a higher level of education. In fact, unlike the female sex workers who often have encountered significant adversities such as childhood trauma or poor family functioning [36, 37], women engaging in compensated dating do not necessarily have such poor experiences [1]. Other research revealed that some women regarded compensated dating as a way to fulfil their sexual excitement, to support their materialistic lifestyle, and to affirm their worth or capability [38]. They also tend to think that compensated dating is a good way to search for passionate relationship and warmth, and prioritize material and pleasure seeking over other concerns [5, 39]. Findings further confirm that women engaging in compensated dating are from a unique population that warrants special attention.

It is important to examine the sexual health of women engaging in compensated dating as previous studies have suggested that compensated dating may be seen as a transitional stage to prostitution in the future [4, 40]. The present study reveals that more than two-thirds of the sample reported having unprotected sex in the past 6 months, and one-tenth of them had been diagnosed with STD before. Findings concur with the literature that women engaging in compensated dating are exposed to significant sexual vulnerabilities [3]. Despite their elevated risk for HIV infection, only less than one-third have ever received HIV testing, and very few (18.6%) have indicated intention to do so in the future. With the rapid development of the Internet technology, women engaging in compensated dating can bargain with their clients through the Internet, which make them feel that they are free to choose the clients they want to meet. Together with the exchange of compassion and companionship, many of them may just consider themselves as “freelance girlfriend” and do not view themselves at-risk of HIV infection. Such findings are worrying and highlight the need for interventions to promote HIV preventive behaviors among this population.

The present study sought to understand factors that would be associated with HIV testing among women engaging in compensated dating in Hong Kong. Consistent with the TPB framework, the present study shows that attitudes, subjective norm, and perceived behavioral control are significantly associated with intention to take up HIV testing. For those women who indicate stronger intention to uptake HIV testing in the future, they are more likely to have possessed a positive attitude towards HIV testing, perceived that their peers were supportive of HIV testing or had previously taken up HIV testing, and had higher sense of control over the act. Findings concur with the previous studies that TPB variables were significant in predicting HIV testing intention in various populations [27–29]. The significant association between perceived behavioral control and HIV testing intention also support the notion that perceived behavioral control is an important concept for behaviors that are characterized as under incomplete volitional control [19]. The present study provided further support to the TPB and extended its utility to HIV testing of women engaging in compensated dating in Hong Kong.

Beattie has pointed out the numerous challenges to assessing HIV service among FSWs [13]. In addition to the TPB model, it is therefore also important to assess how various structural and contextual barriers might exert an influence in one’s intention to take up HIV testing. Findings of the present study show that perceived discrimination from health care workers was the main barrier to HIV testing. Findings corroborate with the extant literature that stigma and perceived discrimination are strong barriers to help-seeking for health care services [41]. Individuals who perceived discrimination from health care providers might be less likely to utilize HIV testing in order to avoid the negative experiences of discrimination and stigmatization. On the other hand, female sex work is a highly stigmatized occupation in the Chinese culture, as sex workers are generally perceived as defying acceptable social norms and roles for women [42]. Studies have shown that FSWs are often subjected to discrimination and social ostracism by the society [43, 44]. To encourage uptake of HIV testing, stigma and discrimination towards sex work and compensated dating need to be reduced.

### Implications for practice

This present study provided important implications for interventions to promote HIV testing among women engaging in compensated dating. The significant association between extended TPB variables and intention to take up HIV testing implies that interventions are needed to promote positive attitudes, subjective norm, and perceived behavioral control. To promote positive attitudes toward HIV testing, education to increase knowledge about benefits of HIV testing is warranted. To promote positive subjective norm associated with HIV testing, encouraging the sharing of experiences among those women who have taken up the HIV testing would be useful to cultivate a supportive culture towards HIV testing. Finally, interventions are needed to enhance sense of personal control over HIV testing. There has been evidence that personal control can be promoted by improving self-efficacy and controllability, which can be enhanced by various techniques such as social modeling, mastery experience, and social persuasion [45].

Furthermore, the significant negative association between perceived discrimination from health care workers and intention to take up HIV testing proposed that discrimination against both sex work and compensated dating should be addressed. The self-stigma of women engaging in compensated dating should also be given attention as it could be a consequence of the internalization of external discrimination [46]. Previous studies have suggested that public education to increase awareness of sex work in the community would be important to reduce discrimination against sex work. Training should also be provided to health care professionals to develop their understanding and acceptance towards women engaging in compensated dating. Policies to ban discrimination towards sex workers among health care professionals are also warranted.

### Limitations

There are several limitations of the present study that should be noted. First, the present study was cross-sectional in nature so the causal relationship between the variables could not be assumed. Second, the study was based on self-report, their intention to take up HIV testing or other related variables might have been overestimated. However, it is important to note that confidentiality and anonymity were assured by using online survey. Third, as there are no validated measures for TPB and barriers to HIV testing, most of the items were self-developed. Some of the constructs assessed in this study did not have satisfactory Cronbach’s alpha (i.e. α < 0.7). Other constructs, e.g. perceived behavioral control, were measured using only one item which may not fully capture the construct. Nevertheless, to ensure that the items were readable and relevant to the subject, a pilot study was conducted among FSWs and all items were deemed relevant and important to the present study. Fourth, self-selection bias might occur that those who agreed to take part might have more positive views towards HIV testing. Fifth, due to the cross-sectional nature of the study, only association between extended TPB variables and intention to take up HIV testing was examined. As suggested by other theories [47], gap between intention and actual behavior could exist. Longitudinal study is needed to examine if the variables under investigation would prospectively predict actual HIV testing behavior in the future. Other factors may also need to be included so to increase its predictability for actual HIV testing behavior. Finally, previous studies have proposed that there might be mediation effects between the TPB variables [21]. Future studies should seek to delineate the interrelationships between the variables using more advanced statistical methods.

## Conclusion

To conclude, the present study demonstrates the applicability of extended TPB in HIV testing intention among women engaging in compensated dating. The importance of perceived discrimination from health care professionals as a barrier was also highlighted. Interventions are needed to promote HIV testing among this population and such interventions should seek to promote positive attitudes towards HIV testing, subjective norm, increase sense of control over HIV testing, and reduce discrimination from the public and staff who provide HIV services.

## Supporting information

S1 File Questionnaire items in Chinese. (DOCX)
