# Supplementary material for: Understanding the barriers and factors to HIV testing intention of women engaging in compensated dating in Hong Kong: The application of the extended Theory of Planned Behavior
Source: PLoS One. 2019 Jun 27;14(6):e0213920. doi: 10.1371/journal.pone.0213920 (PMC6597045; doi:10.1371/journal.pone.0213920)
Supplement: S1 File — (DOCX) [file pone.0213920.s001.DOCX]

做愛滋病測試能讓你安心

(Taking up HIV testing can give you a peace of mind.)

如果感染了愛滋病，做愛滋病測試可以盡早發現病情及接受治療

(If you have HIV, taking up HIV testing allows you to detect HIV and seek early treatment)

對你很重要的人會支持你做愛滋病測試

(The people who are important to you will support you to take up HIV testing.)

你認為有多少會用性交換錢或禮物的香港女性曾經做過愛滋病測試?

(From our understanding, how many women engaging in compensated dating in Hong Kong have received HIV testing?)

做或不做愛滋病測試，完全在你自己的掌握之中

(Taking up HIV testing or not is under your control)

做愛滋病測試會受到工作人員的歧視

(Health care workers will discriminate against you if you take up HIV testing.)

做愛滋病測試讓你感到尷尬

(Taking up HIV testing makes you feel embarrassed)

做愛滋病測試的過程讓你感到焦慮

(The process of HIV testing makes you feel nervous)

等待愛滋病測試結果的過程讓你不安

(You feel anxious when waiting for the HIV testing result)

你害怕可能拿到陽性的測試結果

(You are fear of the possibility of getting a positive result)

你擔心工作人員會問你私隱的問題

(You are worried that workers will ask you very personal questions)

你擔心愛滋病測試結果的保密性

(You are worried about confidentiality of the HIV testing result)

你擔心做愛滋病測試會透露你是性工作者

(You are worried that taking HIV testing will reveal that you have engaged in compensated dating)

你找不到方便做測試的時間

(There isn’t a convenient time for you to do HIV testing.)

做愛滋病測試的地方對你來說不方便

(The venue for HIV testing is not convenient)
